# Chondroitin Sulphate Proteoglycan 4 (NG2/CSPG4) Localization in Low- and High-Grade Gliomas

**DOI:** 10.3390/cells9061538

**Published:** 2020-06-24

**Authors:** Marta Mellai, Laura Annovazzi, Ilaria Bisogno, Cristiano Corona, Paola Crociara, Barbara Iulini, Paola Cassoni, Cristina Casalone, Renzo Boldorini, Davide Schiffer

**Affiliations:** 1 Dipartimento di Scienze della Salute, Scuola di Medicina, Università del Piemonte Orientale (UPO), Via Solaroli 17, 28100 Novara, Italy; marta.mellai@uniupo.it (M.M.); renzo.boldorini@uniupo.it (R.B.); 2 Centro Interdipartimentale di Ricerca Traslazionale sulle Malattie Autoimmuni e Allergiche (CAAD), Università del Piemonte Orientale (UPO), Corso Trieste 15A, 28100 Novara, Italy; 3 Fondazione Edo ed Elvo Tempia Valenta—ONLUS, Via Malta 3, 13900 Biella, Italy; 4 Ex Centro Ricerche/Fondazione Policlinico di Monza, Via P. Micca 29, 13100 Vercelli, Italy; lannov16@gmail.com (L.A.); ilaria.bisogno@gmail.com (I.B.); davide.schiffer@unito.it (D.S.); 5 Istituto Zooprofilattico Sperimentale del Piemonte, Liguria e Valle d’Aosta, Via Bologna 148, 10154 Torino, Italy; cristiano.corona@izsto.it (C.C.); paola.crociara@izsto.it (P.C.); barbara.iulini@izsto.it (B.I.); 6 Dipartimento di Scienze Mediche, Università di Torino/Città della Salute e della Scienza, Via Santena 7, 10126 Torino, Italy; paola.cassoni@unito.it

**Keywords:** NG2/CSPG4, gliomagenesis, neo-angiogenesis, prognosis, immunotherapy, CAR-Ts

## Abstract

Background: Neuron glial antigen 2 or chondroitin sulphate proteoglycan 4 (NG2/CSPG4) is expressed by immature precursors/progenitor cells and is possibly involved in malignant cell transformation. The aim of this study was to investigate its role on the progression and survival of sixty-one adult gliomas and nine glioblastoma (GB)-derived cell lines. Methods: NG2/CSPG4 protein expression was assessed by immunohistochemistry and immunofluorescence. Genetic and epigenetic alterations were detected by molecular genetic techniques. Results: NG2/CSPG4 was frequently expressed in IDH-mutant/1p19q-codel oligodendrogliomas (59.1%) and IDH-wild type GBs (40%) and rarely expressed in IDH-mutant or IDH-wild type astrocytomas (14.3%). Besides tumor cells, NG2/CSPG4 immunoreactivity was found in the cytoplasm and/or cell membranes of reactive astrocytes and vascular pericytes/endothelial cells. In GB-derived neurospheres, it was variably detected according to the number of passages of the in vitro culture. In GB-derived adherent cells, a diffuse positivity was found in most cells. NG2/CSPG4 expression was significantly associated with *EGFR* gene amplification (*p* = 0.0005) and poor prognosis (*p* = 0.016) in astrocytic tumors. Conclusion: The immunoreactivity of NG2/CSPG4 provides information on the timing of the neoplastic transformation and could have prognostic and therapeutic relevance as a promising tumor-associated antigen for antibody-based immunotherapy in patients with malignant gliomas.

## 1. Introduction

Neuron glial antigen 2 (NG2) or chondroitin sulphate proteoglycan 4 (CSPG4) is expressed in the developing and adult central nervous system (CNS). NG2-glia represent the oligodendrocyte precursor cells (OPCs), the most abundant population of endogenous/resident progenitor cells that are able to react to any type of injury and potentially to repopulate areas of lesion hampering and, in the meantime, axon regeneration [1,2,3,4]. NG2-glia can generate astrocytes [5,6] or remain as self-renewing NG2-glia [2]. Embryonic NG2-glia with astrogenic potential migrate and differentiate into astrocytes; alternatively, astrocytes differentiate into mature oligodendrocytes [7]. Besides neural connections [7,8,9,10], NG2-glia represent progenitors that may express NG2/CSPG4 in a short temporal and regional manner [1]. As a matter of fact, in vitro NG2-glia can differentiate into type 2 astrocytes [11,12,13]; under specific cell culture conditions, oligodendrocyte-type 2 astrocyte (O2A) progenitors can self-renew and generate oligodendrocytes, type 2 astrocytes, and even neurons [14,15,16]. Immunotoxic approaches [17] have provided greater supporting evidence compared to conventional studies using transgenic NG2/CSPG4-knockout [18,19]. It is worth considering that NG2-glia may be involved in the origin of gliomas [20,21]. In extra-neural tissues, NG2/CSPG4 expression is important in vascular pericytes and endothelial cells (ECs) [22,23]; a transient expression has been described in macrophages [24].

The protein has a molecular weight of ~250 kDa in its native form and ~300 kDa in the glycosylated state. A large extracellular domain of 2225 amino acids, accounting for 95% of the protein, a transmembrane domain of 25 amino acids, and a short cytoplasmic tail of 76 amino acids [25], are the main structural domains of the core protein. The extracellular domain intervenes in the regulation of the neuronal network [26] and in EC- and pericyte-related mechanisms [27]. The intracellular domain interacts with signal-regulated kinases 1 and 2 (ERK1/2) and protein kinase C-alpha (PKCα) that regulate proliferation, migration, invasion, cytoskeletal reorganization, survival, and chemoresistance [25,28]. The underlying mechanism is the NG2/CSPG4-dependent activation of α3β1 integrin when the two molecules are expressed in the same cell or in two different cells [29].

Chondroitin sulphate (CS) has been previously biochemically and histochemically demonstrated with glycosoaminoglycans (GAGs) in human gliomas [30,31,32,33] and in rat tumors which had been transplacentally induced using *N*-ethyl-*N*-nitrosourea (ENU) [34,35,36]. It has been found in human oligodendroglioma, pilocytic astrocytoma, and, heterogeneously, in glioblastoma (GB). This is consistent with the origin of most gliomas from the subcortical white matter that is rich in OPCs expressing NG2/CSPG4, platelet-derived growth factor alpha (PDGFRα), and oligodendrocyte lineage transcription factor 2 (Olig2) [37]. However, this hypothesis is still a matter of debate [38,39,40]. Alcian blue positivity for CS was detected in isomorphic ENU oligodendrogliomas and in the peripheral parts of polymorphic gliomas [35] in recent findings [41]. Murine oligodendroglioma cells show the basic characteristics of OPCs [20].

In gliomas, NG2/CSPG4 exhibits a cell membrane staining, differing from the cytoplasmic pattern that is typical of resident NG2/CSPG4-positive cells in a normal brain [42], with variable expression. It is co-expressed with PDGFRα, the high levels and gene mutations of which are regarded as features of the proneural subtype of GB [43]. In the latter, NG2/CSPG4 conditions a poor patient survival rate, correlating with the malignancy grade [44,45,46,47].

NG2/CSPG4 triggers tumor initiation, cell proliferation, and neo-vascularization, affecting patients’ clinical outcomes [48,49,50,51]. It regulates the passage to highly vascular gliomas by sequestering angiostatin [52], it conditions chemoresistance by the activation of α3β1 integrin–dependent phosphatidylinositol 3′-kinase (PI3K)-Akt signaling [53], and it promotes a resistance to ionizing radiation with an inverse relationship with apoptosis, demonstrated by its restoration after a small interfering RNA (siRNA) knockdown [45].

NG2/CSPG4 is expressed by immature precursor/progenitor cells and represents a marker for an activated non-quiescent cell status [44]. In the CNS, it marks OPCs, the most important population of cycling cells in the adult CNS [54,55]. The protein contains binding sites for the basic fibroblast growth factor (bFGF) and PDGF-AA [56] and, once blocked, the proliferation of OPCs is inhibited [25]. NG2/CSPG4 expression is mutually exclusive to that of glial fibrillary acidic protein (GFAP) and does not correspond to that of Olig2 [57].

NG2/CSPG4 has a role in blood vessel development both in the normal brain [58] (where blood vessel development is altered in NG2/CSPG4 null tumors [44]) and in the proliferative vessels of malignant gliomas [42,59,60,61] (where ECs are preceded and even guided by migrating pericytes during the organization of the growing vessel wall [23,62]). Basically, NG2/CSPG4 marks mural cells [60]. A tube formation without ECs, but with NG2/CSPG4- and PDGFRβ-positive cells, can even occur [61]. Possibly, the NG2/CSPG4 ectodomain that is shed from pericytes after proteolytic cleavage recruits, at a distance, ECs to sites of angiogenesis, activating β1 integrin. Besides the CNS, NG2/CSPG4 is expressed in mesenchymal stem cells, melanocytes, osteoblasts, smooth muscle (SM) cells, and macrophages [63,64].

In gliomas, NG2/CSPG4 is emerging as a potential tumor-associated antigen (TAA) [65,66,67,68,69,70] for antibody-based immunotherapy, using chimeric antigen receptor-based T cell (CAR-Ts) therapy in patients with GB [47]. As it conditions cell proliferation, chemoresistance, and survival, the possibility of exploring its theranostic properties has been greatly emphasized.

The aim of the present study was to analyze the role of NG2/CSPG4 in relation to malignant progression and prognosis in a series of 61 adult human gliomas of different molecular subtypes and in nine GB-derived cell lines At the protein level, NG2/CSPG4 expression was evaluated by immunohistochemistry (IHC) in tumor tissues and by immunofluorescence (IF) in cell lines.

Associations of NG2/CSPG4 with isocitrate dehydrogenase 1 and 2 (IDH1/2), p53 tumor suppressor protein (TP53), and telomerase reverse transcriptase (TERT) promoter mutations, epidermal growth factor receptor (EGFR) gene amplification, O^6^-methylguanine-DNA methyltransferase (MGMT) promoter hypermethylation, 1p/19q co-deletion, and alpha-thalassemia/mental retardation syndrome X-linked (ATRX) status were tested as well.

Based on these findings, we wanted to evaluate the biological rationale for considering NG2/CSPG4 as a druggable therapeutic target using CAR-Ts in patients with GB.

## 2. Materials and Methods

### 2.1. Brain Tumor Specimens

This study included 61 adult gliomas (seven grade II and two grade III astrocytomas, 14 grade II and eight grade III oligodendrogliomas, and 30 GBs) which were collected at the Units of Pathology of Città della Salute e della Scienza/University of Turin (Turin, Italy) and Maggiore della Carità Hospital/University of Eastern Piedmont (Novara, Italy) (Table 1).

Patients underwent partial or total resection. Surgical tumor specimens were formalin fixed and paraffin embedded (FFPE), and cut into 5 μm-thick serial sections. The histologic diagnosis was in agreement with the current World Health Organization (WHO) guidelines [71].

Healthy nervous tissue was obtained from the brain autopsies of patients who had died from vascular encephalopathies.

Surgical tumor specimens from five IDH-wild type GBs were split into three consecutive fragments. The first one was FFPE and cut into 5 μm-thick sections. The second one was minced and enzymatically dissociated for expansion in culture, whereas the third fragment was stabilized in RNAlater^®^ solution (Thermo Fisher Scientific Inc., Waltham, MA, USA) and then stored at −80 °C for molecular genetics.

### 2.2. Ethics Statement

Human brain specimens were obtained and used in compliance with the local institutional review board and the Committee on Human Research and with the ethical human subject principles of the World Medical Association Declaration of Helsinki Research. Written informed consent was obtained from patients after Institutional Ethics Committee approval.

### 2.3. In Vitro Cultures

Fresh surgical tissue from five IDH-wild type GB patients was processed for in vitro culture, as described [72]. Five cell lines were isolated as neurospheres (NS) (TO-02 NS, TO-03 NS, TO-04 NS, TO-07 NS, and TO-09 NS) in Dulbecco’s modified Eagle’s medium (DMEM)/F-12, supplemented with 20 ng/mL epidermal growth factor (EGF) and 10 ng/mL bFGF for the NeuroSphere Assay (NSA). Four cell lines were developed as adherent cells (TO-02 AC, TO-04 AC, TO-07 AC, and TO-09 AC) in DMEM with 10% fetal bovine serum (FBS). Both cultures were maintained in a 5% O_2_ and 5% CO_2_ humidified atmosphere. The NS abilities of self-renewal, multipotency, clonogenicity, and tumorigenicity were assessed, as already reported [72].

Cell line authentication from the matched primary tumor was verified by short tandem repeat profiling. Experiments were carried out on cell lines from passages 10–40.

In vitro cultures were monthly checked for mycoplasma contamination before experimental use (e-Myco^TM^ Mycoplasma PCR Detection kit, iNtRON Biotechnology, Seongnam-si, Korea).

### 2.4. IHC

Besides haematoxylin and eosin (H&E) staining, IHC was performed using a Ventana Full BenchMark^®^ XT automated immunostainer (Ventana Medical Systems, Inc., Tucson, AZ, USA). The ultraView^TM^ Universal DAB Detection Kit (Ventana) was the detection system. Heat-induced epitope retrieval was obtained with cell conditioning solution (CC1-Tris based EDTA buffer, pH 8.0, Ventana). Negative controls were obtained by the omission of the primary antibody. The primary antibodies are listed in Table 2. Nestin, Sox2, and MSel1 were considered as representative of GB stem cells (GSCs)/progenitors [73,74].

Double immunostainings for GFAP/Nestin, GFAP/ATRX, and CD34/Iba-1 were performed with the ultraView^TM^ Universal Alkaline Phosphatase Red Detection Kit (Ventana).

NG2/CSPG4’s immunoreactivity was evaluated using a semi-quantitative system for the percentage of positive cells, the staining intensity, and type of distribution (focal or diffuse) in five randomly selected microscopic high-power fields (HPF), at a ×400 magnification, per tumor section.

The frequency of glioma-associated microglia/macrophages (GAMs) was quantified as previously described [75].

IHC for ATRX was used as a surrogate marker for the mutation status of the *ATRX* gene. The quantification methods for ATRX have been already reported [76].

### 2.5. IF

IF was performed on all nine GB-derived cell lines. Cells were fixed for 20 min with 4% paraformaldehyde at room temperature, rinsed three times with phosphate-buffered saline (PBS), and blocked/permeabilized for 30 min with 1X phosphate-buffered saline (PBS), containing 2% of the appropriate serum and 0.1% Triton X-100. Then, they were stained with the primary antibodies which are indicated with ° in Table 2. Negative controls were obtained by omitting the primary antibody. Alexa Fluor^®^ 488-AffiniPure goat anti-rabbit IgG and Alexa Fluor^®^ 594-AffiniPure rabbit anti-mouse IgG (Jackson ImmunoResearch Laboratories, Inc., West Grove, PA, USA) were used as secondary antibodies. Cell nuclei were counterstained with 4′,6-diamidino-2-phenylindole (DAPI). Images were acquired on a Zeiss Axioskop fluorescence microscope (Carl Zeiss, Oberkochen, Germany) equipped with an AxioCam MRc5 digital camera coupled to an imaging system (AxioVision Release 4.5, Zeiss).

The frequency of NG2/CSPG4+ cells was quantified by calculating the mean number of positive cells in five randomly selected HPF at a ×400 magnification.

Following the same procedure, IF was also assessed on tissue sections from ten IDH-wild type GBs and five IDH-mutant/1p19q-codel oligodendrogliomas.

### 2.6. Molecular Genetics

Genomic DNA (gDNA) from the FFPE tumor samples was isolated using the QIAamp DNA Mini Kit (Qiagen NV, Venlo, The Netherlands).

The search for mutations in *IDH1* (exon 4) (GenBank sequence NM_005896), *IDH2* (exon 4) (GenBank sequence NM_002168), the *TERT* gene promoter region (GenBank accession no. NM_198253), and the *TP53* (exons 4–8) genes (GenBank sequence NM_000546) was performed by Sanger direct sequencing on an ABI^®^ 3130 Genetic Analyzer (Thermo Fisher Scientific, Inc.) [77]. The BigDye Terminator v1.1 Cycle Sequencing Kit (Thermo Fisher Scientific, Inc.) was used. Data were collected by the Sequencing Analysis v.5.3.1 software (Thermo Fisher Scientific, Inc.). The reported nucleotide and amino acid numbering was relative to the transcription start site (+1), corresponding to the A of the ATG on the GenBank reference sequences. The sequence variant nomenclature was in agreement with the current Human Genome Variation Society guidelines (http://varnomen.hgvs.org/).

The 1p/19q chromosomal status was assessed by Multiplex Ligation-dependent Probe Amplification (MLPA) using the SALSA-MLPA Kit P088-C2 (lot numbers 0608-0112) (MRC-Holland, Amsterdam, The Netherlands), according to the manufacturer’s instructions [78]. After capillary electrophoresis (CE), data were collected by the GeneMapper v4.0 software (Thermo Fisher Scientific, Inc.) and analyzed using Coffalyser v140721.1958 software (MRC-Holland).

Allelic imbalances in the chromosomal regions 9p, 10q, and 17p were assessed by loss of heterozygosity (LOH) analysis and fragment analysis [72]. The *EGFR* gene amplification status (GenBank accession no. NM_005228) was analyzed as described [72].

Quantitative methylation specific-PCR (MS-PCR), followed by fragment analysis and CE, was used to determine the *MGMT* promoter hypermethylation status (GenBank accession no. NM_002412). The primer sequences and amplification conditions for MS-PCR were previously reported [79].

### 2.7. Statistical Methods

Associations between the categorical variables were evaluated using 2 × 2 contingency tables by the two-tailed Fisher’s exact test. Pearson’s correlation coefficient was used to examine the relationship between NG2/CSPG4 immunoreactivity and Ki-67/MIB-1 and Sox2 labelling indices (LIs).

Overall survival (OS) was defined as the time between histologic diagnosis and the patient’s death or last follow-up (FU). Patients who were alive at their last FU were considered as censored events. Survival curves were estimated using the Kaplan–Meier method and differences were compared by the log-rank test (Mantel–Cox). For the survival analysis, the cases were dichotomized into the following categories: negative-NG2/CSPG4 expression cases (≤ 1% positive tumor cells) and positive-NG2/CSPG4 expression cases (> 1% positive tumor cells with focal or diffuse immunoreactivity).

Analysis was carried out by SPSS v25.0 software (SPSS Inc., Chicago, IL, USA). *p*-values < 0.05 were considered statistically significant.

## 3. Results

### 3.1. IHC

A total of 61 grade II-IV gliomas were studied. In the healthy nervous tissue, NG2/CSPG4 was expressed in the cytoplasm of neurons, whereas it was not found in glial cells or ECs. It was absent or not detectable in rarely visible vascular pericytes.

In gliomas, reactive astrocytes, some tumor cells, vascular pericytes, ECs, normal neurons, and macrophages expressed NG2/CSPG4 in the cytoplasm and/or on the cell membrane.

**Figure 1:**
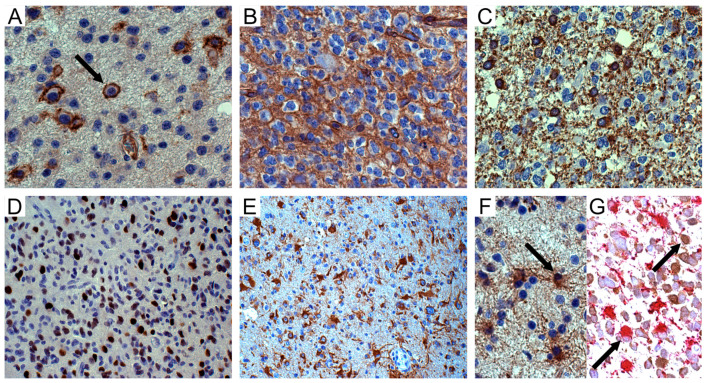
NG2/CSPG4 immunohistochemistry in oligodendroglial tumors. (**A**) WHO grade II oligodendroglioma. Isolated tumor cells (arrow) with NG2/CSPG4-positive cell membranes; DAB, original magnification (OM) ×400. (**B**) WHO grade III oligodendroglioma. NG2/CSPG4-positive area with honeycomb appearance; DAB, OM ×400. (**C**) *Id.* Isolated CNPase-positive tumor cells in infiltration area; DAB, OM ×400. (**D**) *Id*. Sox10-positive tumour cells; DAB, OM ×200. (**E**) *Id.* NG2/CSPG4-positive reactive astrocytes inside the tumor; DAB, OM ×200. (**F**) *Id.* GFAP-positive reactive astrocytes (arrow) intermingled with GFAP-negative tumor cells; DAB, OM ×400. (**G**) *Id.* GFAP-positive (alkaline phosphatase red) and mIDH1^R132H^-negative reactive astrocytes (arrow) intermingled with mIDH1^R132H^-positive (DAB) and GFAP-negative tumor oligodendrocytes (arrow); OM ×400. WHO, World Health Organization; DAB, 3,3′-*Diaminobenzidine*.

In 13/22 (59.1%) IDH-mutant/1p19q-codel oligodendrogliomas, tumor cells expressed NG2/CSPG4, either focally or diffusely, with a slight prevalence in grade III tumors (6/8, 75%) compared to grade II tumors (7/14, 50%) (Table 1) (Figure 1A,B), mainly in infiltration areas. Minigemistocytes and gliofibrillary oligodendrocytes (GFOC) were negative.

Scattered NG2/CSPG4-positive cells expressed 2′,3′-cyclic nucleotide 3′ phosphodiesterase (CNPase), either on the cell membranes and processes or in the cytoplasm of isolated round cells, mainly in infiltration areas toward the normal cortex (Figure 1C). Sox10 was variably expressed in tumor cells (Figure 1D).

NG2/CSPG4 was regularly expressed in reactive astrocytes, either outside or entrapped inside the solid tumor (Figure 1E), forming in GB large areas composed of GFAP-positive spindle-shaped cells or gemistocytes with long processes on vessels. In oligodendroglial tumors, reactive astrocytes were distinguishable from mIDH1^R132H^-positive and GFAP-negative tumor cells based on their GFAP expression in the cytoplasm (Figure 1F) and lack of mIDH1^R132H^ immunoreactivity (Figure 1G).

**Figure 2:**
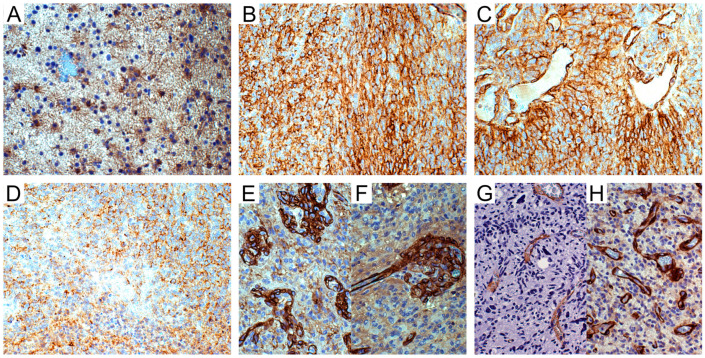
NG2/CSPG4 immunohistochemistry in astrocytic tumors. (**A**) WHO grade II astrocytoma. NG2/CSPG4-negative tumor cells and -positive reactive astrocytes; DAB, original magnification (OM) ×200. (**B**) IDH-wild type glioblastoma (GB). Area of NG2/CSPG4-positive cells; DAB, OM ×200. (**C**) *Id.* NG2/CSPG4-positive tumor cells and reactive astrocytes oriented on vessels; DAB, OM ×200. (**D**) *Id.* NG2/CSPG4-negative area of necrosis; DAB, OM ×200. (**E**) *Id.* NG2/CSPG4-positive vascular pericytes in microvascular proliferations; DAB, OM ×200. (**F**) *Id.* NG2/CSPG4-positive pericytes in a glomerulus with sprout; DAB, OM ×200. (**G**) *Id.* α-SMA-positive pericytes in infiltration area; DAB, OM ×200. (**H**) *Id.* PDGFRβ-positive pericytes in hyperproliferative area; DAB, OM ×200. WHO, World Health Organization; IDH, isocitrate dehydrogenase 1/2; DAB, 3,3’-*Diaminobenzidine*.

In grade II-III astrocytomas, tumor cells were almost negative (Figure 2A). In IDH-wild type GBs, 18/30 (60%) cases were negative, 3/30 (10%) cases were focally positive, and 9/30 (30%) were diffusely positive (Figure 2B–D).

Through the analysis of the semi-serial sections, NG2/CSPG4 immunoreactivity was mutually exclusive from that of GFAP in tumor cells but not in reactive astrocytes. No correlation was found with Nestin expression that was much more diffusely distributed; NG2/CSPG4-positive cells could be either Nestin-positive or Nestin-negative. In GBs, most CD163-positive macrophages showed NG2/CSPG4 immunopositivity.

The NG2/CSPG4 immunoreactivity according to the different molecular subtypes is reported in Table 3.

### 3.2. Relationship between NG2/CSPG4 Expression and Tumor Neo-Vasculature

NG2/CSPG4 was detected in the vascular pericytes or ECs of normal blood vessels or quiescent tumor blood vessels neither in low- nor in high-grade gliomas (LGGs and HGGs, respectively). By contrast, it was strongly expressed in the cytoplasm of the vascular pericytes and ECs of newly proliferated tumor vessels (microvascular proliferations (MVPs) and glomeruli) (Figure 2E,F). Vascular pericytes were revealed by α-SM actin (α-SMA) and PDGFRβ immunoreactivity (Figure 2G,H). Compared to NG2/CSPG4, α-SMA was positive in a lower number of pericytes, whereas PDGFRβ was detected in ECs and pericytes in a higher number.

### 3.3. IF

A total of fifteen tissue sections (from IDH-wild type GBs and IDH-mutant/1p19q-codel oligodendrogliomas) and nine GB-derived cell lines were analyzed by IF. In the former, the distribution pattern of NG2/CSPG4 was similar to the immunohistochemical findings.

**Figure 3:**
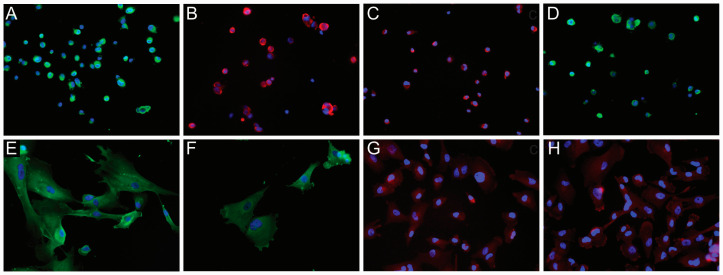
Immunofluorescence (IF) analysis of NG2/CSPG4 expression in glioblastoma (GB)-derived human cell lines. IF of a GB-derived neurosphere cell line for (**A**) NG2/CSPG4 (green), original magnification (OM) ×200; (**B**) Nestin (red), OM ×200; (**C**) MSel1 (red), OM ×200; (**D**) Notch-2 (green), OM ×200. IF staining of an adherent cell line for (**E**) NG2/CSPG4 (green), OM ×400; (**F**) GFAP (green), OM ×400; (**G**) β-III Tubulin (red), OM ×200; (**H**) Galactocerebroside C (red), OM ×200. Nuclei were counterstained with DAPI (blue). DAPI, *4′,6-diamidino-2-phenylindole.*

In the cell lines, most of the GB-derived NS showed a strong NG2/CSPG4 positivity around the nucleus (Figure 3A). The expression of NG2/CSPG4 increased, as for the staining intensity and number of positive cells, from the early (10–20) to late passages (21–40). The mean percentage of NG2/CSPG4-positive cells at ×400 HPF varied from 75.8% (25/33) for the former to 84.9% (45/53) for the latter. In NS, NG2/CSPG4 was co-expressed with Nestin, Sox2, MSel1, and Notch-2. Nestin and Sox10 were intensely positive in all NSs (in the cytoplasm and nucleus, respectively). Sox2 was detected in the nucleus, whereas MSel1 was found in both nucleus and cytoplasm; Olig2 and Notch-2 were expressed in the cytoplasm (Figure 3B–D).

GB-derived ACs showed a diffuse NG2/CSPG4 positive staining in the cytoplasm, mainly around the nucleus (Figure 3E). Besides NG2/CSPG4, they expressed differentiation markers such as GFAP, β-III Tubulin, and galactocerebroside (GalC) with a cytoplasmic staining (Figure 3F–H). MSel1 was mainly found in the nucleus, whereas Notch-2 was variably expressed in the cytoplasm.

### 3.4. Molecular Genetics

*TERT* promoter mutations were detected in 20 out of 41 (48.8%) gliomas. The mutation rate was 16/26 (61.5%) in IDH-wild type GBs and 4/15 (26.7%) in LGGs. All mutations were c.−124C>T (C228T) single nucleotide substitutions.

Point mutations at codons Arg132 of the *IDH1* gene and Arg172 of the *IDH2* genes were identified in 25 out of 60 (41.7%) gliomas. The missense mutation c.395G > A (p.Arg132His) accounted for 23/25 (92%) and c.516G > T (p.Arg172Ser) and c.515G > A (p.Arg172Lys) accounted for 1/25 (4%) each.

The mutation rate of the *TP53* gene was equal to 2 out of 4 (50%) in anaplastic astrocytomas and to 3 out of 18 (16.7%) in IDH-wild type GBs. All mutations detected in the *TERT*, *IDH1/2*, and *TP53* genes were somatic changes.

*EGFR* gene amplification occurred in 19/45 (42.2%) malignant gliomas. The *MGMT* promoter hypermethylation was detected in 19 out of 46 (41.3%) tumors.

Malignant gliomas showed LOH on 10q in 42 out of 46 (91.3%) cases and LOH on 9p in 35 out of 46 (76.1%) cases. LOH on 17p was detected in all TP53-mutant astrocytic gliomas.

Finally, IDH-wild type GBs and oligodendroglial tumors all retained ATRX protein expression in the nucleus, whereas it was lost in 6 out of 9 (66.7%) LG astrocytomas.

The frequency of the above reported molecular changes with respect to the NG2/CSPG4 immunoreactivity is described in Table 3.

### 3.5. Relationship between NG2/CSPG4 and Clinical and Molecular Features

The immunoreactivity of NG2/CSPG4 was not associated with patient age, gender, or anatomic location (all *p* > 0.05, Fisher’s exact test). It increased with the histologic malignancy grade in both astrocytic and oligodendroglial tumors but without statistical significance.

The comparison between NG2/CSPG4 and Ki-67/MIB-1 LIs showed a positive correlation neither in LGGs nor in GBs (*p* > 0.05, Pearson’s test). NG2/CSPG4-positive cells could be either mitotically active or not. Accordingly, in GBs, no correlation was found between NG2/CSPG4 and Sox2 LIs, the latter having previously been described as a marker of cell proliferation for this tumor type [80].

NG2/CSPG4 immunoreactivity was significantly associated with *EGFR* gene amplification (*p* = 0.0005, Fisher’s exact test) in malignant gliomas and with 1p/19q-codeletion in oligodendroglial tumors (*p* = 0.0297, Fisher’s exact test) (Table 3).

### 3.6. Survival Analysis

The relationship between NG2/CSPG4 immunoreactivity and overall survival OS was analyzed in two tumor sub-groups, including 18 oligodendroglial tumors (ten grade II and eight grade III) and 24 astrocytic tumors (seven grade II, two grade III, and 15 GBs). For the survival analysis, the cases were dichotomized into positive (diffuse or focal NG2/CSPG4 immunoreactivity) and negative ones.

For 19 cases (four oligodendrogliomas and 15 GBs), clinical data and FU were not available. Survival analysis of the tumor sub-group of 15 GB patients with recorded FUs was not possible because among the three NG2/CSPG4-positive cases, one died after nine months and two were still alive at 12 months.

**Figure 4:**
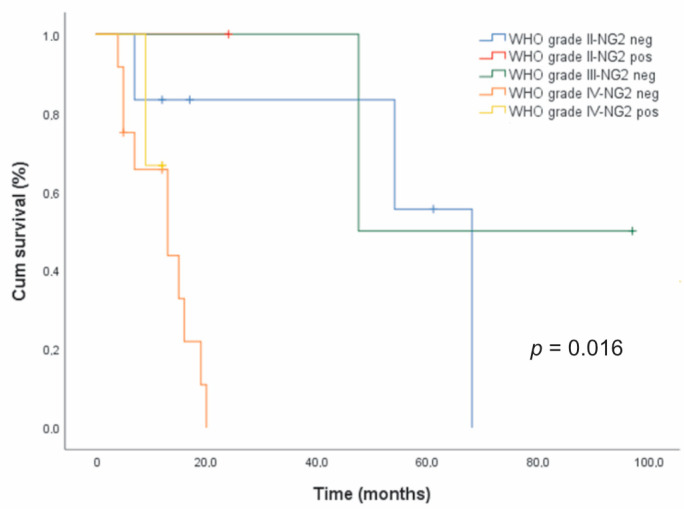
Relationship between NG2/CSPG4 expression and survival in glioma patients. Kaplan–Meier survival curves for overall survival (OS) with respect to NG2/CSPG4 immunoreactivity after patient stratification for the histologic grade in 24 patients with astrocytic gliomas.

In astrocytic tumors, the Kaplan–Meier survival analysis revealed a shorter median survival time for NG2/CSPG4-positive cases (*n* = 4, 16 months) compared to NG2/CSPG4-negative ones (*n* = 20, 19 months), but the difference was not statistically significant (data not shown). Censored cases were 3/4 (75%) for the former and 6/20 (30%) for the latter. However, NG2/CSPG4 immunoreactivity was significantly associated with a poor prognosis (*p* = 0.016, log-rank test) after stratification for the malignancy grade (Figure 4).

In oligodendrogliomas, NG2/CSPG4 immunoreactivity was correlated with a worse prognosis but this was without statistical significance (data not shown). The median survival time was 75 months for NG2/CSPG4-positive cases (*n* = 11) and 97 months for NG2/CSPG4-negative cases (*n* = 7). Censored cases were 9/11 (81.8%) for the former and 6/7 (85.7%) for the latter. After stratification for the histologic grade, grade III oligodendrogliomas, expressing either diffuse or focal NG2/CSPG4 immunoreactivity, showed the worst prognosis (*p* > 0.05, log-rank test) (data not shown).

## 4. Discussion

In the present study, we analyzed the role of NG2/CSPG4 in relation to the origin, progression, and prognosis of 61 adult gliomas. Compared to low-grade tumors, malignant gliomas show an increased and aberrant NG2/CSPG4 overexpression that correlates with a worse prognosis. The positive immunostaining of reactive astrocytes is one of the most important findings.

The NG2/CSPG4 immunoreactivity is diffusely detectable in >75% of anaplastic IDH-mutant/1p19q-codel oligodendrogliomas and in >40% of IDH-wild type GBs. Conversely, it is absent or barely detectable in IDH-mutant or IDH-wild type low-grade astrocytomas. The increased NG2/CSPG4 overexpression in malignant tumors, mainly in infiltration areas, is in agreement with previous findings [81,82]. In oligodendrogliomas, the NG2/CSPG4 immunoreactivity reveals a the honeycomb appearance, i.e., the oligodendroglial component, but also reactive astrocytes.

The subcellular location of NG2/CSPG4 at the protein level could be related to the different functions of the protein domains. The positive staining of the cytoplasm in neurons could be referred to as the intracellular domain of NG2/CSPG4. Conversely, the membranous distribution in tumor cells and in reactive astrocytes is probably due to the transmembrane and extracellular domains. Its variable expression in GB may also be due to the intra-tumor heterogeneity [83,84].

**Figure 5:**
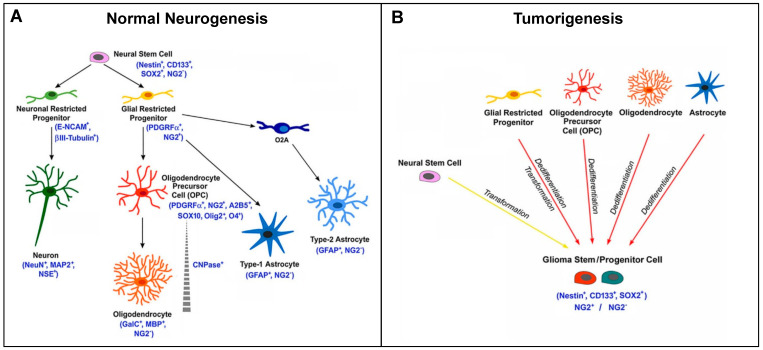
Scheme of NG2/CSPG4 expression in CNS cytogenesis and gliomagenesis. (**A**) Scheme representing the expression of the NG2 marker during normal neurogenesis and the different cell fates of NG2-glia. Canonically, NG2-glia (NG2+ glial restricted progenitors) has the capability to proliferate and differentiate into oligodendrocyte precursor cells (OPCs), then giving rise to oligodendrocytes in the immature and mature brain. However, NG2-glia can also differentiate into astrocytes. Additionally, in vitro, NG2-glia can differentiate into type-2 astrocytes through the O2A progenitor cells. (**B**) NG2+ cells can also be considered as potential cells for the origin of malignant glioma. During tumorigenesis, the glioma stem cell is believed to derive from the transformation of neural stem cells or from the dedifferentiation and transformation of NG2+ glial restricted progenitors, OPCs, or mature cells (astrocytes and oligodendrocytes). The arising tumor cells show potential for self-renewal and express markers associated with both stem and progenitor cell types [85].

NG2/CSPG4 marks OPCs in approximately 60% of the oligodendrogliomas. During cytogenesis, NG2/CSPG4 is expressed from a certain stage of development until terminal differentiation in adult oligodendrocytes. NG2/CSPG4-positive or NG2/CSPG4-negative tumors could originate from the transformation of precursors/progenitors at different timepoints (either before expressing NG2/CSPG4, during its expression, or after its loss) (Figure 5) [85]. They could correspond to tumors with different maturation degrees, proliferation rates, and survival times, according to their early or late derivation.

Taking into account the low number of cases, we suggest a prevalence of NG2/CSPG4 expression in malignant gliomas and a tendency toward a better prognosis for NG2/CSPG4-negative tumors. As a matter of fact, in the survival analysis, NG2/CSPG4 immunoreactivity was significantly associated with a worse prognosis in astrocytic gliomas after patient stratification for the histologic grade. A similar tendency has been observed in oligodendrogliomas, but the difference in the median survival time between NG2/CSPG4-positive and NG2/CSPG4-negative cases is not statistically significant. The number of censored cases is too high with respect to the temporal values of our collection. This consideration is also valid for the tumor sub-group of GBs.

The most important observation is the NG2/CSPG4 expression in reactive astrocytes. As their frequency varies in the different surgical samples (mainly in GB where they can be found in a very high number), they could be responsible for the variable expression of NG2/CSPG4. In the present study, we confirm an inter-grade NG2/CSPG4 variability in gliomas and its correlation with the malignancy grade, in agreement with previous findings [44,45,46,47,86]. An intra-grade comparison was not possible because of the poor number of cases.

The positive NG2/CSPG4 immunostaining in reactive astrocytes could be responsible for the focal positivity in tumors but does not affect the general interpretation because of the dismal significance of the reactive astrocytes [87]. As a matter of fact, the survival analysis, if limited to cases characterized by diffuse immunoreactivity, did not reveal any significant correlation with prognosis. To explain the widespread NG2/CSPG4 expression, we can speculate that tumors may originate from the transformation of progenitors after acquiring NG2/CSPG4 expression.

Reactive astrocytes can be found either at early onset or at a later stage, both in peritumor tissue and entrapped in the tumor. They can appear as hyperplastic Nestin-positive and GFAP-positive cells with a small and globoid cytoplasm without processes or as hypertrophic GFAP-positive and Nestin-negative cells with a larger cytoplasm and thick and long processes [88,89]. The advancing tumor incorporates reactive astrocytes either as round cells or as GFAP-positive multipolar, spindle, or gemistocytic cells, with long processes on vessels. Their morphological aspect could depend on their age and on the invasion speed of the tumor.

Reactive gliosis around gliomas is a typical finding [90], but it is also known that reactive astrocytes in gliomas have a different meaning in comparison to gliosis in other pathological conditions [89]. However, in the neocortex after injury, NG2/CSPG4-positive cells are not the major source of reactive astrocytes [91]. In addition, progenitors may originate protoplasmic astrocytes of the cortex [90,92] before expressing NG2/CSPG4, consistently with the capability of NG2/CSPG4-positive precursors to originate reactive astrocytes [93,94]. In our experience, tumor astrocytes do not express NG2/CSPG4 in these areas.

The fate of reactive astrocytes entrapped in the tumor still remains unknown. In our opinion, they remain visible for a long time in the proliferative tumor due to their GFAP positivity and NG2/CSPG4 expression, undergoing the pathoplastic influence from tumor cells, mainly in relation to vessels. The double staining for GFAP/ATRX demonstrates that reactive astrocytes retain ATRX expression in the nucleus (i.e., with *ATRX*-wild type), whereas in diffuse astrocytomas, tumor astrocytes are ATRX-mutant [76]. Different explanations can be suggested: (i) they are converted into tumor cells, (ii) they remain as vestiges, or (iii) they are destroyed.

NG2/CSPG4 immunoreactivity only partially overlaps with the expression of CNPase and PDGFRβ; vascular pericytes express PDGFRβ, NG2/CSPG4, and α-SMA only in a late stage. Their increase corresponds with the growth of newly formed vessels (MVPs and glomeruli) and, therefore, they can be considered a reliable marker of neo-angiogenesis.

By IF, we could confirm that NG2/CSPG4 positivity marks immature progenitor cells that represent the majority of cells under in vitro culture conditions. NG2/CSPG4-negative cells are possibly those with a lower grade of commitment, i.e., with features closer to stem cells. The increase in NG2/CSPG4-positive cells in late passages may indicate the aging of the in vitro culture, i.e., the increase of cell differentiation. The greater number of NG2/CSPG4-positive cells in NS derived from tumors with the highest cell proliferation index and poor survival time is in line with this interpretation. In AC, the subcellular location around the nucleus, rather than on the cell membrane, might suggest the involvement of the intracellular domain in differentiation [11,14], controlling, at the same time, cell proliferation and pericyte migration.

There is an apparent inconsistency between the predominant NG2/CSPG4 positivity of NS and the negativity of most tumor cells in GB. We analyzed a panel of primary cell lines, isolated by IDH-wild type GBs, that we supposed to be derived directly from the transformation of stem cells or NG2/CSPG4-negative early progenitors. We can hypothesize that: (i) tumors mainly develop from NG2/CSPG4-negative cells, in spite of the co-existence of positive ones under in vitro cultures, (ii) the latter have lost the capability of expressing NG2/CSPG4 during the malignant transformation, or (iii) the microenvironment triggers tumor cells towards stemness [95] and, therefore, NG2/CSPG4 negativity.

In astrocytomas, tumor astrocytes may derive from OPCs and lose NG2/CSPG4 positivity by differentiation; on this point, previous speculations about astrocyte derivation from OPCs become relevant [2,3,4,6,11,12,13]. Oligodendrogliomas might derive from NG2/CSPG4-expressing precursors or from not yet or no longer expressing it, so that the different progression potentials could compensate for the survival evaluation.

The heterogeneous expression of NG2/CSPG4 in GB-derived NS indicates that they are probably composed of both GSCs and progenitors. Our data suggest that in NS, no more than 10%–15% of the cells are true GSCs that do not yet express NG2/CSPG4; the remaining 85%–90% of the cells would be composed of NG2/CSPG4-expressing progenitors. By contrast, the ACs all express NG2/CSPG4 as they are already committed. The majority of the NG2/CSPG4-positive cells would proliferate as progenitors and not as GSCs [90].

The highest NG2/CSPG4 expression in oligodendrogliomas, if compared to the most frequent astrocytic tumors, would suggest that oligodendroglial cells exhibit the hallmarks of progenitors rather than of neural stem cells (NSCs). This finding supports the origin of the tumor from the transformed (NG2/CSPG4-expressing) progenitors, rather than from the transformed NSCs, that may contribute to the better outcome.

Finally, the significant association between NG2/CSPG4 immunoreactivity and *EGFR* gene amplification (a well-known negative prognostic marker) in both astrocytic and oligodendroglial tumors is in line with previous findings [45]. It corresponds to the unfavorable prognosis of NG2/CSPG4-positive cases revealed by the survival analysis. NG2/CSPG4 could play a functional role in the EGFR-PI3K-AKT signaling pathway by enhancing the activation of the tyrosine kinase domain of *EGFR* and the proliferative capability of GB cells [38,95].

All the above findings on NG2/CSPG4 in gliomas may have a translational relevance. NG2/CSPG4 could be regarded as a new biomarker of malignancy and poor prognosis, and a promising TAA for a CAR-Ts therapy in patients with malignant gliomas [96,97].

## Figures and Tables

**Table 1:** NG2/CSPG4 immunoreactivity according to the molecular subtype.

Molecular Subtype	WHO Grade	Patients *(n)*	Mean Age (Years) and Range	NG2/CSPG4 Immunoreactivity *n (%)*		
				Focal	Diffuse	Total
O, IDH-mutant/1p19q-codel	II	14	45 (23–75)	3 (42.9)	4 (57.1)	7 (50)
AO, IDH-mutant/1p19q-codel	III	8	57 (31–80)	0 (0)	6 (100)	6 (75)
A, IDH-mutant	II	6	41 (20–65)	1 (100)	0 (0)	1 (14.3)
A, IDH-wild type	II	1	46	0 (0)	0 (0)	0 (0)
AA, IDH-mutant	III	1	59	0 (0)	0 (0)	0 (0)
AA, IDH-wild type	III	1	53	0 (0)	0 (0)	0 (0)
GB, IDH-wild type	IV	30	60 (25–86)	3 (25)	9 (75)	12 (40)

**Abbreviations:** WHO, World Health Organization; NG2/CSPG4, neuron glial antigen 2/chondroitin sulphate proteoglycan 4; O, oligodendroglioma; AO, anaplastic oligodendroglioma; A, astrocytoma; AA, anaplastic astrocytoma; GB, glioblastoma; IDH, isocitrate dehydrogenase; mt, mutant; wt, wild type.

**Table 2:** List of primary antibodies used for immunohistochemistry and immunofluorescence.

Antibody (Clone)	Source	Dilution	Code	Company
Ki-67 (MIB-1) *	Mouse	1:100	M7240	Dako
GFAP	Mouse	1:200	M0761	Dako
GFAP °	Rabbit	1:200	Z0334	Dako
mIDH1^RI32H^ (H09) *	Mouse	1:20	DIA H09	Dianova
ATRX *	Rabbit	1:400	HPA001906	Sigma-Aldrich
NG2/CSPG4 *	Rabbit	1:50	NBP1-89682	Novus Biological
CD34 *	Mouse	Pre-diluted	790-2927	Ventana Medical Systems, Inc.
Iba-1 *	Rabbit	1:500	#019-19741	Wako Chemicals
CD68 (KP-1) *	Mouse	Pre-diluted	790-2931	Ventana Medical Systems, Inc.
CD163 (MRQ-26)	Mouse	Pre-diluted	760-4437	Ventana Medical Systems, Inc.
Sox2 *^,^°	Mouse	1:50	MAB2018	R&D Systems
Sox10 (EP268) IVD	Rabbit	1:100	AC-0237A	Epitomics
Olig2 °	Rabbit	1:200	AB9610	Millipore
α-SMA (1A4) *	Mouse	Pre-diluted	760-2833	Ventana Medical Systems, Inc.
PDGFRβ (Y92) *	Rabbit	1:50	ab32570	Abcam
CNPase *	Rabbit	1:200	NBP1-85999	Novus Biologicals
Nestin *^,^°	Mouse	1:200	MAB5326	Dako
βIII-Tubulin *^,^°	Mouse	1:250	MAB1637	Chemicon
GalC *^,^°	Mouse	1:200	MAB342	Chemicon
MSel1 *	Mouse	1:350	−	Kind gift
Notch-2 *^,^°	Rabbit	1:200	sc-5545	Santa Cruz Biotech.

**Abbreviations:** GFAP, glial fibrillary acidic protein; IDH1, isocitrate dehydrogenase 1; ATRX, alpha-thalassemia/mental retardation syndrome X-linked; NG2/CSPG4, neuron glial antigen 2/chondroitin proteoglycan 4; Iba-1, ionized calcium-binding adapter molecule 1; Sox2, sex determining region Y-box 2; PDGFRβ, platelet-derived growth factor receptor β; GalC, Galactocerebroside C; MSel1, mouse monoclonal anti-suppressor of Lin-12-like protein (*C. elegans*). **Note:** * Heat-induced epitope retrieval required; ° Tested by immunofluorescence.

**Table 3:** Association of common molecular alterations with NG2/CSPG4 immunoreactivity in gliomas.

Molecular Marker	Overall Mutation Rate *(n, %)*	Alteration Rate in NG2/CSPG4 + Cases *(n, %)*	Alteration Rate in NG2/CSPG4 − Cases *(n, %)*	*p*-Value
TERT promoter mutations	20/41 (48.8)	8/20 (40)	12/21 (57.1)	NS
IDH1/2 mutations	25/60 (41.7)	10/24 (41.7)	15/36 (41.7)	NS
TP53 mutations	5/22 (22.7)	2/8 (25)	3/14 (21.4)	NS
EGFR gene amplification	19/45 (42.2)	11/13 (84.6)	8/32 (25)	0.0005
MGMT promoter methylation	19/46 (41.3)	9/21 (42.9)	10/25 (40)	NS
1p/19q co-deletion	22/34 (64.7)	13/15 (86.6)	9/19 (47.3)	0.0297
LOH 9p	35/46 (76.1)	18/24 (75)	17/22 (77.3)	NS
LOH 10q	42/46 (91.3)	22/24 (91.7)	20/22 (90.9)	NS
LOH 17p	5/22 (22.7)	2/8 (25)	3/14 (21.4)	NS

**Abbreviations:** NG2/CSPG4, neuron glial antigen 2/chondroitin sulphate proteoglycan 4; TERT, telomerase reverse transcriptase; EGFR, epidermal growth factor receptor; LOH, loss of heterozygosity; IDH, isocitrate dehydrogenase; MGMT, O^6^-methylguanine-DNA methyltransferase; TP53, tumor protein p53; NS, not significant.
